# Intercostal Cryoneurolysis for Chest Wall Pain in Patients With Vertebral Osteomyelitis

**DOI:** 10.7759/cureus.102010

**Published:** 2026-01-21

**Authors:** Renato Abu Hana, Ruben G Ortiz Cordero, Oswaldo A Guevara Tirado, Vinicius Adami Vayego Fornazari, Grit A Adler

**Affiliations:** 1 Department of Radiology, University of Florida College of Medicine Jacksonville, Jacksonville, USA

**Keywords:** chest wall pain, douleur neuropathique en 4 questions, intercostal cryoneurolysis, interventional radiology, neuropathic pain, vertebral osteomyelitis

## Abstract

Intercostal cryoneurolysis (IC) is an image-guided technique that provides prolonged analgesia through targeted cold-induced axonal degeneration. While it is most commonly used for post-traumatic, postoperative, and malignancy-related chest wall pain, its role in infection-associated pain syndromes remains poorly described. We report the case of a 53-year-old man with a history of vertebral osteomyelitis complicated by abscess formation who developed chronic right-sided dermatomal chest wall pain and sought an alternative to long-term pharmacologic therapy. The patient initially underwent an ultrasound-guided diagnostic intercostal nerve block, followed by computed tomography-guided IC. This report discusses patient selection, procedural technique, and potential complications associated with IC for intractable chest wall pain.

## Introduction

Chronic chest wall pain represents a heterogeneous clinical entity, with etiologies ranging from musculoskeletal disorders to neuropathic and visceral causes [1,2]. In rare cases, spinal infections such as vertebral osteomyelitis or paraspinal abscesses may manifest as radicular or dermatomal chest wall pain, posing diagnostic and therapeutic challenges [3-5]. Pain associated with spinal infection is thought to result from inflammatory changes and compression of adjacent nerve roots [3,4]. Case reports have described an association between inflammatory masses or osseous destruction and band-like, nonpleuritic chest pain [4]. Additionally, epidural infections may present with radicular pain corresponding to the dermatomal distribution of the affected vertebral level [3].

Because the intercostal nerves arise from the anterior rami of T1-T12 and innervate the thoracic wall and parietal pleura, thoracic spine pathology can produce band-like, dermatomal chest wall pain [1]. Standard management strategies often rely on pharmacologic therapies, including opioids and neuromodulatory agents, which may be poorly tolerated or undesirable for long-term use due to concerns regarding dependence [2].

Intercostal cryoneurolysis (IC) is a minimally invasive, image-guided technique that induces reversible axonal injury through cold application, resulting in prolonged analgesia while preserving nerve integrity [1]. It has been described in the management of post-thoracotomy pain, rib fractures, chest wall malignancies, and intercostal neuralgia [1,6,7]. Additionally, Cha et al. demonstrated that IC may reduce inpatient opioid use and hospital length of stay in selected chest wall pain populations [6]. However, its use in infection-associated neuropathic chest wall pain remains poorly described in the literature.

## Case presentation

A 53-year-old man with multiple comorbidities, including insulin-dependent diabetes mellitus and paraplegia secondary to a prior deep T4-T8 paraspinal abscess with chronic osteomyelitis, was admitted for a urinary tract infection (UTI) and referred to interventional radiology for evaluation of a one-year history of chronic, intractable right-sided chest wall pain in the T4-T7 dermatomal distribution. The pain was described as burning and shock-like, associated with painful muscle spasms, and had been poorly controlled with medical therapy, requiring chronic opioid use. At the time of evaluation, there was no evidence of active thoracic osteomyelitis.

A structured pain assessment using the Douleur Neuropathique en 4 questions (DN4) questionnaire yielded a score of 6/10, consistent with a neuropathic pain component [8]. The DN4 is a validated 10-item screening tool that differentiates neuropathic from nonneuropathic pain based on symptom descriptors and bedside sensory testing, with scores ≥4/10 supporting a neuropathic pain diagnosis [8] (see the Appendix). At the time of evaluation, the patient reported partial symptom control with oral oxycodone (10 mg) and diazepam (10 mg) but expressed concern regarding long-term opioid use. After discussion of treatment options, informed consent was obtained to proceed with an ultrasound-guided right intercostal nerve block as a short-term therapeutic measure and diagnostic step to assess candidacy for a more durable intervention such as cryoneurolysis.

Laboratory studies obtained prior to the procedure demonstrated mild anemia (hemoglobin 10.9 g/dL), normal white blood cell count (6.09 10³/µL), and mildly elevated international normalized ratio (INR: 1.2). These findings were reviewed and were not deemed contraindications to the procedure.

**Figure 1:**
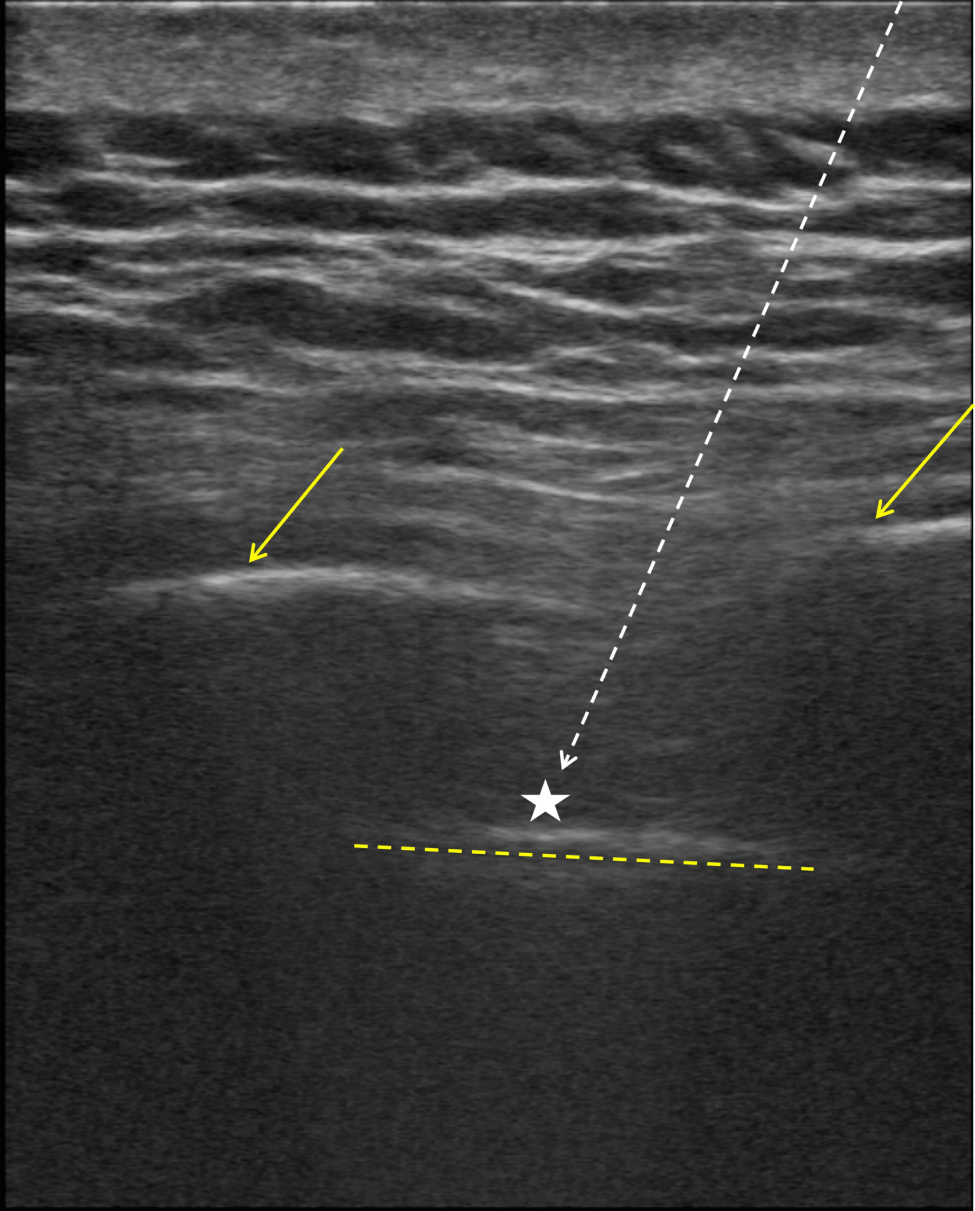
Ultrasound-guided intercostal nerve block. A 25-gauge spinal needle (limited needle visualization; white dashed arrow) is advanced with a caudal to cranial angulation into the subcostal groove (star), positioned just superior to the pleura (yellow dashed line) and between adjacent ribs (solid yellow arrows)

For the ultrasound-guided right intercostal nerve block, the patient was positioned in the left lateral decubitus position. Under ultrasound guidance, a 25-gauge spinal needle was advanced into the plane between the innermost intercostal muscle and the pleura at the T5-T6 level, where 3 mL of 0.5% ropivacaine was injected (Figure 1), followed by additional injections of 3 mL at the adjacent T4-T5 and T6-T7 intercostal spaces, for a total volume of 9 mL of 0.5% ropivacaine. The patient tolerated the procedure without complications and subsequently reported complete resolution of symptoms, supporting the intercostal nerves as the primary pain generators and consistent with previously described diagnostic approaches for neuropathic chest wall pain [1,6].

Two weeks after the nerve block, the dermatomal pain and muscle spasms recurred while the patient was receiving extended antibiotic therapy for a multidrug-resistant UTI in the hospital’s rehabilitation unit. Interventional radiology was reconsulted, and the patient was scheduled for computed tomography (CT)-guided right IC. Laboratory studies at that time demonstrated persistent mild anemia (hemoglobin 10.7 g/dL), a normal white blood cell count (5.05 × 10³/µL), and a minimally elevated INR (1.4), which were again not considered contraindications to the procedure.

After informed consent was obtained, the procedure was performed with the patient in the prone position under moderate sedation using 3 mg of midazolam and 150 mcg of fentanyl, with standard pre- and postsedation assessment and continuous cardiorespiratory monitoring. Localizing CT images were obtained. The skin and subcutaneous tissue were anesthetized with 15 mL of 2% lidocaine.

**Figure 2:**
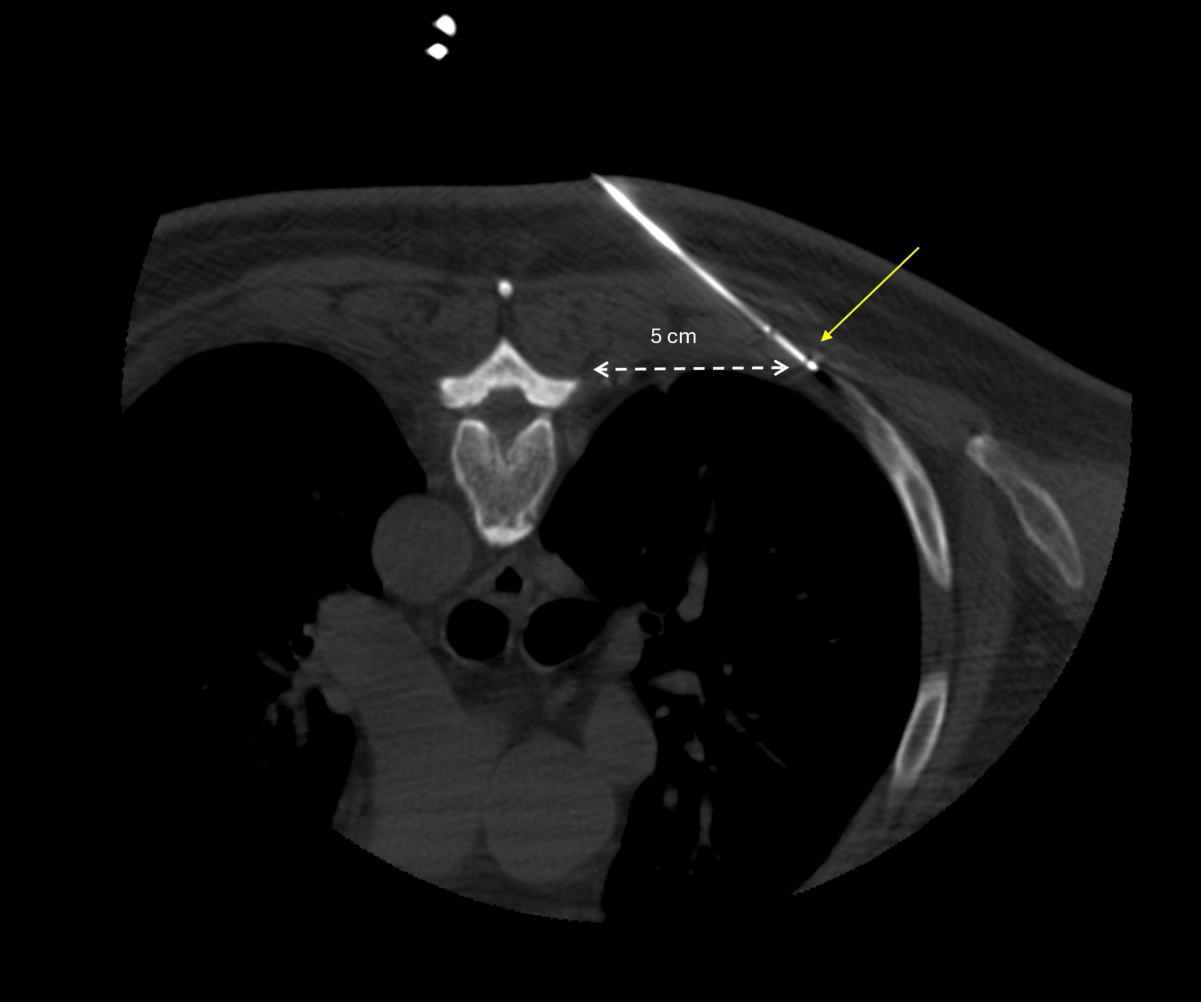
Intraprocedural axial computed tomographic image of the cryoablation probe placement (yellow arrow), at the level of the affected rib with ablation zone distancing 5 mm away from the subcostal groove and 5 cm away from the thoracic nerve roots

**Figure 3:**
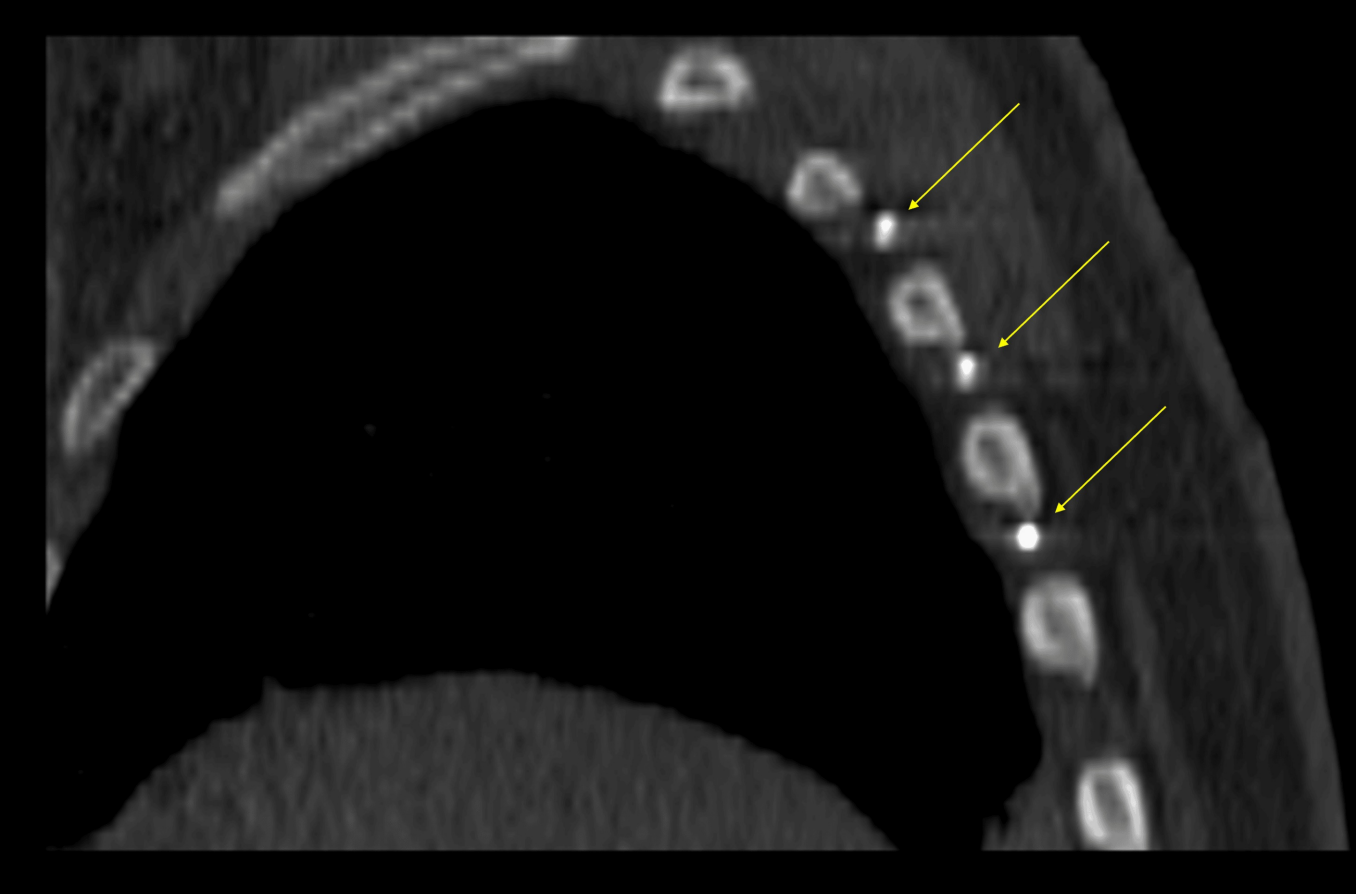
Intraprocedural sagittal computed tomographic image showing three cryoablation probes (arrows) within T4-T7 intercostal spaces

**Figure 4:**
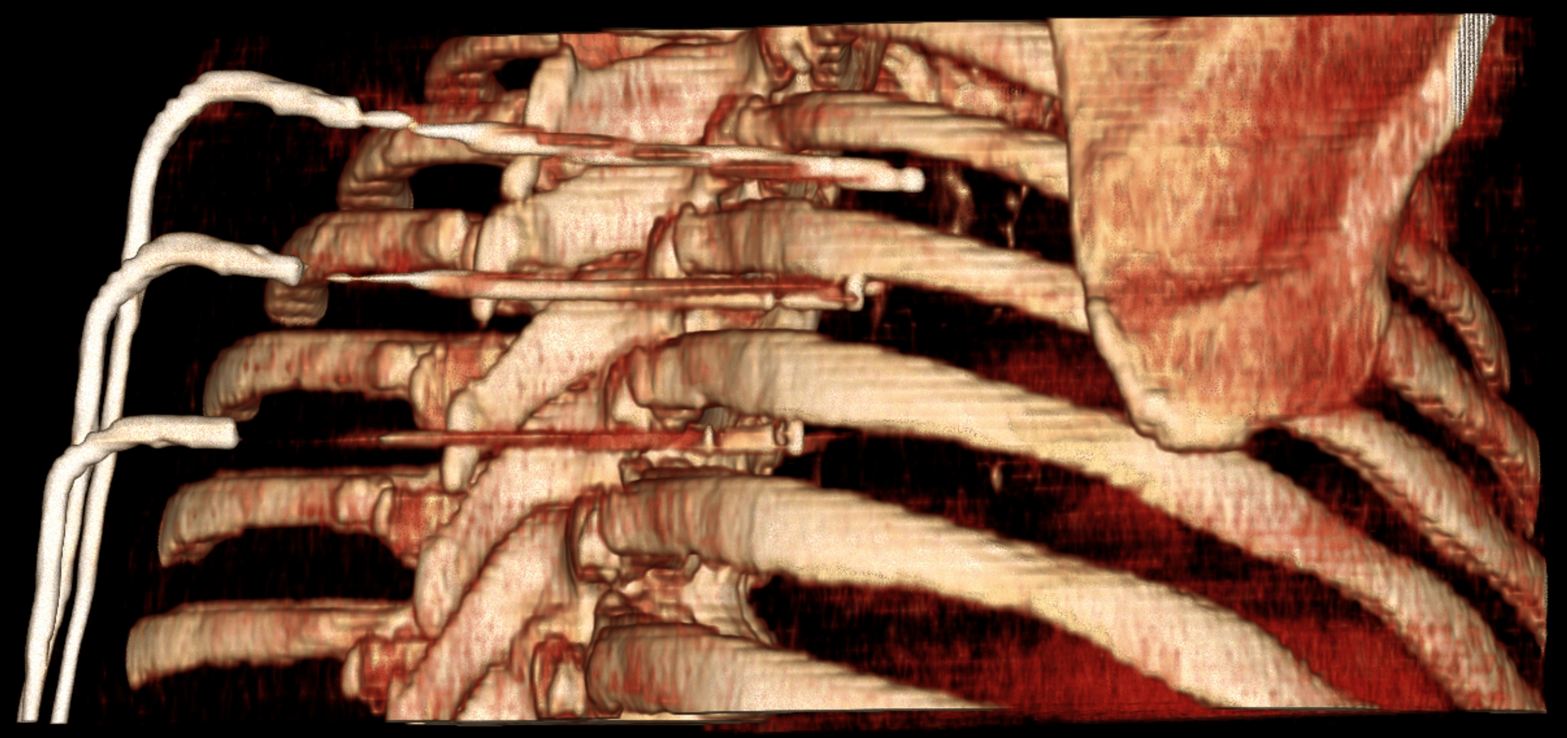
Multiplanar CT reconstruction demonstrating multiple cryoprobes from T4 to T7 intercostal levels CT: computed tomography

Under CT guidance, three 17-gauge ICESEED™ 1.5 cryoablation probes (Boston Scientific, Boston, MA) were inserted 5 cm from the right neuroforamen into the T4-5, T5-6, and T6-7 intercostal spaces and 5 mm from the subcostal groove to avoid vascular injury with the probe (Figures 2-4). Once probes were placed, two alternating freeze-thaw cycles were performed, with the first cycle being an eight-minute ablation at 100%, followed by an active three-minute thaw, and the second cycle being a three-minute ablation at 100%, followed by a three-minute active thaw.

Intermittent CT imaging after the initial eight-minute freeze demonstrated appropriate ice-ball formation. Postcryoneurolysis CT showed no acute complications, including pneumothorax, lung injury, or hematoma. The patient tolerated the procedure well and remained in the department for one hour for routine postprocedural monitoring.

On short-term inpatient follow-up evaluation, the patient reported significant improvement in his muscle spasms after several days. The patient was later discharged to the rehabilitation unit to continue his ongoing antibiotic treatment for his UTI.

## Discussion

Chest wall pain secondary to infection, such as vertebral osteomyelitis, is uncommon and may result from nerve root irritation or inflammatory extension into adjacent neural structures [3-5]. In such cases, accurate identification of a neuropathic pain component is essential to guide targeted interventions. The DN4 questionnaire is a validated and widely used screening tool for neuropathic pain and provides objective support for procedural decision-making in this case [8]. More specifically, a DN4 score of ≥4/10 has good diagnostic performance, with about 83% sensitivity and 90% specificity for neuropathic pain, and can correctly classify pain in approximately 86% of patients [8].

Analgesic strategies for chronic neuropathic chest wall pain can include multimodal pharmacotherapy with nonsteroidal anti-inflammatory drugs, opioids, and gabapentin [1,2]. However, long-term use of these medications can lead to dependence, tolerance, or side effects [1]. Regional nerve blocks can also be used for pain management, but their analgesic effect is often temporary [6,7]. While our patient’s dermatomal chest pain was being managed adequately by oral pain medications, his desire to find a longer lasting and nonaddictive solution in conjunction with the chronicity of his pain led to the consideration of an intercostal block followed by IC.

IC has demonstrated efficacy in managing various forms of neuropathic chest wall pain while reducing opioid consumption and improving quality of life [1,6,7]. Complications are low, and reported adverse events include chest wall hematomas, pneumothorax, chest wall laxity and numbness, neuroma or pseudohernia formation, and postoperative neuralgia [1,6,8]. If performed using appropriate technique and under imaging guidance, complications are minimized because IC does not damage important structures, such as the perineurium, epineurium, vessels, or bones [1,6,7].

In our patient, the use of CT guidance to position the probe within the subcostal groove while maintaining a safe distance from critical structures, together with real-time monitoring of ice-ball formation, allowed the procedure to be completed without any immediate or delayed complications. Furthermore, our case highlights that, when combined with structured neuropathic pain assessment and confirmatory diagnostic nerve blocks, IC was associated with symptomatic improvement [1,6].

## Conclusions

IC is an image-guided, minimally invasive technique that has been used for the treatment of post-traumatic, postoperative, malignant, and idiopathic neuropathic chest wall pain. This report describes a rare case in which IC provided a nonpharmacological pain control alternative for chronic dermatomal chest wall pain presumed to be a sequela of prior vertebral osteomyelitis following appropriate neuropathic pain assessment and confirmatory diagnostic nerve block. Although limited to a single patient, this case suggests that IC may provide sustained analgesia in selected patients with infection-associated neuropathic chest wall pain. Further studies and long-term follow-up are needed to better characterize the durability of pain relief after IC in this population.
